# Ethical considerations in the UK-Nepal nurse recruitment: Nepali nurses’ perspectives

**DOI:** 10.1177/09697330241305574

**Published:** 2025-01-07

**Authors:** Animesh Ghimire, Yunjing Qiu, Mamata Sharma Neupane, Purushottam Ghimire

**Affiliations:** 2541 Monash University; Sustainable Prosperity Initiative Nepal; 1994 University of Technology Sydney; 475236 Chitwan Medical College; Sustainable Prosperity Initiative Nepal

**Keywords:** Colonialism, exploitation, ethics, global health inequities, nurse migration

## Abstract

**Background:**

The global migration of nurses from resource-constrained to affluent nations raises complex ethical concerns, often rooted in historical power imbalances and neocolonial legacies. The Nepal-UK Memorandum of Understanding (MoU) on nurse recruitment, while presented as a solution to workforce shortages, exemplifies this complex dynamic, prompting critical questions about its implications for individual nurses and the healthcare systems involved.

**Aim:**

This qualitative study explored the ethical complexities and dilemmas associated with the Nepal-UK nurse recruitment Memorandum of Understanding (MoU). This bilateral agreement has sparked debate about its potential impact on both individual nurses and the healthcare systems of Nepal and the UK.

**Research Design:**

A qualitative exploratory design utilizing semi-structured interviews was employed. Data were analyzed using reflexive thematic analysis.

**Participants and Research Context:**

Twelve Nepali nurses from two private hospitals in Kathmandu, Nepal, participated in the study.

**Ethical Considerations:**

The study was approved by the Nepal Health Research Council. All participants provided informed consent and were assured of confidentiality and anonymity.

**Results:**

Four themes emerged from the data: (1) The lingering legacy of colonialism casts a shadow on the Nepal-UK relationship, raising concerns about potential exploitation and unequal power dynamics. (2) Nepali nurses grapple with the ethical dilemmas of pursuing personal dreams while acknowledging their responsibilities towards their communities and Nepal’s healthcare system. (3) The MoU’s claims of ethical recruitment are scrutinized, with nurses questioning its fairness and sustainability. (4) The agreement is challenged as a potential band-aid solution that may perpetuate global health inequities rather than fostering a genuine partnership.

**Conclusions:**

The Nepal-UK MoU, while offering opportunities for individual nurses, also raises alarms about brain drain, exploitation, and the perpetuation of global health disparities. The study underscores the urgent need for a paradigm shift in international nurse recruitment practices, prioritizing genuine partnership, equitable distribution of benefits, and sustainable solutions that address the root causes of healthcare workforce challenges in both source and destination countries.

## Introduction

The global migration of nurses, particularly the movement of skilled healthcare professionals from resource-constrained countries to high-income countries (HICs), is a complex phenomenon deeply intertwined with historical power imbalances and contemporary economic disparities.^1^ This pattern is propelled by a confluence of push and pull factors. In source countries, often characterized by limited career prospects, inadequate working conditions, and insufficient healthcare infrastructure, nurses are driven to seek opportunities abroad.^2,3^ Conversely, HICs grappling with their own healthcare workforce challenges actively recruit these nurses, offering attractive salaries, improved working conditions, and the promise of enhanced professional growth.^4,5^ This migration is often further driven by HICs efforts to uphold their healthcare standards and mitigate workforce shortages despite having significantly higher nurse-to-population ratios compared to those in the source countries.^6,7^ For instance, there are 9.2 nurses per 1000 in the UK^8^ compared to 3.5 nurses per 1000 in Nepal.^9^ This stark contrast, translating to fewer than 115,900 registered nurses serving a population of over 30 million in Nepal, underscores the severity of the healthcare workforce crisis in the country.^10^ Recruiting nurses from countries with existing shortages may be perceived as a readily available and cost-effective solution despite the ethical concerns surrounding the potential exploitation of these healthcare professionals and the impact on their home countries’ already strained healthcare systems.^11,12^ This intricate dynamics surrounding nurse migration highlight the necessity of assessing its ethical implications. This analysis is crucial not only for the individual nurses confronted with challenging decisions but also for the healthcare infrastructure and communities they depart from and integrate into.

## Background

Amidst a global nursing shortage, Nepal grapples with the stark reality of brain drain as a significant number of its healthcare professionals seek opportunities abroad.^2,13^ It is important to highlight that Nepal, designated as a lower-middle-income country (LMIC) in 2020 with a gross national income (GNI) per capita of $1,090,^14^ encounters considerable difficulties in delivering sufficient healthcare to its citizens.^15^ Therefore, this exodus is particularly alarming considering Nepal’s placement on the World Health Organization (WHO) “red list” due to critical health workforce shortages and alarming nurse-to-population ratios.^9,16^ While the ethical implications of this brain drain have been widely acknowledged in the literature,^17–19^ there remains a critical gap in understanding how specific policies, such as bilateral agreements between countries, contribute to or mitigate these ethical challenges. Nepal’s position as a top contributor of nurses to the UK’s workforce, despite being on the WHO’s “red list,” raises serious concerns about the fairness and sustainability of such recruitment practices.^20,21^ This study aims to address this gap by specifically examining the ethical complexities and dilemmas associated with the Nepal-UK nurse recruitment Memorandum of Understanding (MoU). This bilateral agreement has sparked debate about its potential impact on both individual nurses and the healthcare systems of Nepal and the UK.

This context calls for a deeper analysis of the challenges facing the UK’s healthcare system, which have led to an increased dependence on foreign recruitment. The difficulties in retaining the nursing workforce, driven by insufficient pay and challenging working conditions, have resulted in a systemic vulnerability that requires external solutions.^22–24^ Furthermore, the reliance on international recruitment raises questions about the UK’s commitment to addressing these internal challenges and investing in its domestic healthcare workforce.^25^ The ethical implications of this approach, particularly for source countries like Nepal, cannot be ignored.

The ongoing global nursing shortage and the ethical dilemmas associated with international recruitment have led to the emergence of the Nepal-UK Memorandum of Understanding (MoU) as a contentious initiative.^26^ This MoU seeks to address the critical challenge of meeting workforce demands while simultaneously maintaining adherence to established ethical standards in the recruitment process.^26,27^ This bilateral agreement, with its stated emphasis on managed migration and reciprocal goals, purports to offer a more ethically sound approach to international nurse recruitment.^26^ However, its efficacy in achieving this delicate balance remains contested. Despite its protective measures, it maintains power imbalances and worsens global health disparities.^20^ The ethical Code of Practice for international recruitment of healthcare workers prohibits active recruitment from countries experiencing severe workforce shortages unless a government-to-government agreement exists.^20^ Hence, this MoU raises questions about whether the agreement truly prioritizes the needs of Nepali nurses and their communities or merely serves as a convenient solution to the UK’s staffing crisis.

The International Council of Nurses (ICN) has expressed concerns about the global shortage of nurses, projected to reach a deficit of nine million by 2030, and the potential for unethical recruitment practices in the context of increased international migration.^28^ The ICN emphasizes the importance of ethical recruitment, advocating for countries to train and retain their own nurses while adhering to WHO standards for international recruitment.^28^ The ICN position statement on international career mobility and ethical nurse recruitment highlights the need for comprehensive regulation, access to full employment opportunities, freedom of movement, non-discrimination, good faith contracting, equal pay, safe work environments, effective orientation and supervision, and freedom of association.^29^ ICN also calls for national self-sufficiency, urging governments to focus on developing a sustainable national nursing workforce and implementing robust workforce planning systems.^29^ These concerns underscore the importance of examining the ethical implications of the Nepal-UK MoU and its potential impact on both individual nurses and the healthcare systems of both countries.

## Aims

This study aims to answer the following research question: How do Nepali nurses perceive the ethical implications of the Nepal-UK nurse recruitment Memorandum of Understanding, particularly in relation to historical power dynamics, individual aspirations versus collective well-being, and the potential for neocolonial exploitation in the context of global health inequities?

## Methods

### Study design

This research employed a qualitative exploratory design^30^ to explore the ethical considerations surrounding the Nepal-UK nurse recruitment agreement. This design was deemed most appropriate due to the limited existing research examining the ethical dimensions of this MoU from Nepali nurses’ perspective. An exploratory approach allowed for in-depth exploration of nurses’ perceptions, experiences, and concerns, generating rich qualitative data to illuminate the complex ethical landscape surrounding this policy initiative. The study adhered to the Consolidated Criteria for Reporting Qualitative Research (COREQ) guidelines to ensure transparency and rigor in the research process.^31^

### Setting and participants

The study was conducted within two urban private hospitals in Kathmandu, Nepal. Despite our best efforts, we could not recruit participants from rural hospitals, as none of the nurses registered for the study. This may indicate a potential divide between nurses’ experiences in urban and rural settings. Whether this lack of participation stems from hesitancy, a heavier workload in rural settings, or other factors specific to rural contexts warrants further exploration.

Participants were recruited through purposive sampling, ensuring they met the following inclusion criteria: (1) a minimum of 2 years of professional nursing experience, (2) demonstrated knowledge and awareness of the MoU, and (3) willingness to voluntarily participate in the study. This sampling approach aimed to select participants with the necessary professional background and contextual understanding to provide rich and nuanced insights into the ethical dimensions of the MoU.

### Data collection

Data were collected through individual, semi-structured interviews conducted by two authors (AG and MSN). To recruit participants, flyers containing a QR code for self-registration were disseminated across various departments within the two selected hospitals. The interviews were audio-recorded, enabling participants to articulate their views comfortably in their preferred language, either Nepali or English. Subsequently, the authors (AG and MSN) transcribed all audio recordings verbatim. During transcription, personal identifiers were replaced with codes to ensure participant anonymity. Nepali responses were meticulously translated into English to facilitate comprehensive data analysis. Each interview typically lasted between 40 and 45 minutes and was scheduled at a convenient time and location for the participants, ensuring their comfort and minimizing any disruptions to their work schedule. The semi-structured interview guide (Table 1), informed by the research aims, provided a flexible framework for exploring participants’ perceptions and experiences regarding the ethical dimensions of the Nepal-UK nurse recruitment MoU.

**Table 1.** Semi-structured interview question guide.

Question no.	Interview questions
1	Can you describe your understanding of the Nepal-UK Memorandum of Understanding (MoU) on nurse recruitment?
2	What are your thoughts on the ethical implications of the MoU, particularly in terms of fairness and potential exploitation of Nepali nurses?
3	How do you perceive the power dynamics between Nepal and the UK in the context of this agreement? Do you feel there’s an imbalance?
4	In your opinion, does the MoU perpetuate any historical patterns of colonialism or neocolonialism? If so, how?
5	What are your personal motivations for considering migration to the UK as a nurse? How do these motivations intersect with your sense of responsibility towards Nepal’s healthcare system?
6	Do you see any potential benefits for Nepal’s healthcare system from this MoU, such as skills transfer or remittances?
7	What are your concerns about the potential impact of nurse migration on Nepal’s healthcare system, particularly in terms of brain drain and access to care?
8	Do you believe the MoU adequately addresses the ethical concerns surrounding international nurse recruitment, such as transparency, informed consent, and protection against exploitation?
9	In your view, what steps could be taken to ensure a more equitable and sustainable approach to international nurse recruitment, both within the context of the MoU and more broadly?
10	How do you envision the future of nursing in Nepal, considering the ongoing trend of migration and the potential impact of agreements like the MoU?

### Data analysis

The first author (AG) transcribed all audio-recorded interviews verbatim and translated them into English where necessary. To ensure accuracy and mitigate potential bias, the second and the third authors (YQ and MSN) independently reviewed a selection of these transcripts. Adhering to Braun and Clarke’s^32^ guidelines for reflexive thematic analysis, the research team embarked on an iterative process of data familiarization, code generation, and theme development. Initially, open coding was employed to identify salient excerpts and assign descriptive codes. These codes were then systematically organized into broader categories based on shared conceptual meanings, facilitating the emergence of overarching themes through ongoing discussion and consensus within the research team.

To ensure the findings’ trustworthiness, member checking was conducted by sharing preliminary results with participants and inviting their feedback to validate the interpretations and ensure an accurate representation of their experiences. Further enhancing rigor, two researchers (YQ and MSN) independently coded a portion of the data, with any discrepancies resolved through collaborative discussion. The entire analytical journey, encompassing the evolution of codes, categories, and themes, was documented in an audit trail, promoting transparency and replicability (Table 2).

**Table 2.** Illustrative examples of the coding process.

Data extract	Initial codes	Categories	Subthemes	Themes
“It’s like history repeating itself [..] First, it was our Gurkha soldiers fighting their wars; […] into whenever it suits them?” (P11)	Power imbalance, Unfairness, Resource extraction	Unequal exchange, Exploitation, One-sided benefit	Power asymmetry, Neocolonial undertones	From Gurkhas to Nurses: Echoes of Colonialism in Nepal-UK Relations
“It’s a constant battle between my head and my heart. [..] at the thought of leaving […].” (P2)	Conflicting desires, Moral dilemma, Duty vs. ambition	Personal aspirations vs. collective responsibility, Ethical conflict, Guilt	Internal struggle, Moral responsibility, Sense of duty	Dreams and Dilemmas: Navigating the Ethical Complexities of Nurse Migration
“This agreement feels like a step in the right direction. For too long, Nepali nurses have been undervalued and underpaid […].” (P3)	Recognition, Fairness, Positive change	Valuing skills, Addressing inequity, Hope for improvement	Empowerment, Acknowledgment of worth, Progress	Fair Trade or Foul Play? Ethics of International Nurse Recruitment
“The MoU is a band-aid solution. It might temporarily alleviate the UK’s staffing crisis, [..] feel compelled to leave.” (P10)	Short-term fix, Systemic issues, Unsustainable	Superficial solution, Root causes unaddressed, Perpetuating problems	Lack of systemic change, Ineffective long-term, Questionable sustainability	Band-Aid Solutions or Genuine Partnership? The MoU’s Role in Addressing Global Health Inequities

In line with the approach advocated by Rahimi and Khatooni,^33^ the study aimed for code and thematic saturation rather than data saturation, acknowledging the diverse experiences and perspectives reflected in the data. While no new codes or themes emerged during the analysis of the final interviews, it was recognized that individual narratives might continue to vary with the inclusion of additional participants. This approach prioritized the depth and richness of the analysis over the sheer volume of data collected.

### Rigor and reflexivity

We recognize that qualitative research is inherently subjective and that researcher reflexivity is crucial for ensuring the trustworthiness of the findings.^34^ Throughout this study, we engaged in critical self-reflection to examine our own backgrounds, assumptions, and potential biases. As migrant nurses (AG and YQ), we were acutely aware of the challenges healthcare professionals face in source countries and the ethical complexities of leaving one’s homeland for opportunities in high-income countries. The author (MSN), a nursing professional and an academic in the Nepalese setting and a non-migrant, brought the insider perspective, allowing us to connect deeply with the study participants, fostering trust, and facilitating open and honest dialogue. However, we also remained mindful of the potential for our own experiences to influence data interpretation. To mitigate this, we employed strategies such as independent coding and collaborative discussion with the author (PG), a Nepalese health policy expert, to ensure the rigor and validity of our findings.

### Ethical considerations

This study received ethical approval from the Nepal Health Research Council (Registration Number: 431/2024). All participants provided written informed consent before participating and were assured confidentiality and anonymity throughout the research process.

## Results

### Participants characteristics

The 12 participants in this study were all female registered nurses with an age range of 27–47 years, a mean age of 33.42 years, and an average of 7.5 years of professional experience. This exclusively female sample reflects the prevailing gender dynamics in Nepal’s nursing landscape, where nursing is predominantly perceived as a female-dominated field. This observation is further substantiated by a report from the International Labor Organization indicating that the nursing workforce in Nepal is entirely female.^35^ The participants represented a diverse range of clinical specialties, including critical care, emergency medicine, maternity, medical-surgical, operating room, cardiology, neurology, psychiatry, geriatrics, and administration. Their positions ranged from registered nurses to nurses in charge, reflecting varied levels of responsibility and experience within the nursing profession. Furthermore, the participants demonstrated a range of educational backgrounds, with seven nurses holding a Master’s degree in Nursing and the remaining five possessing a Bachelor’s degree in Nursing. This diversity in educational qualifications adds another layer of depth to the sample, potentially influencing their perspectives on career progression and the opportunities presented by the MoU.

While recognizing the importance of intersectionality in research, this study did not explicitly collect data on participants’ race or ethnicity. In the context of Nepal, urban hospitals primarily serve a relatively homogenous population. These categories were not deemed essential for understanding the core research questions focused on the ethical dimensions of the MoU. However, we acknowledge that future research could explore the potential influence of ethnicity and caste within the Nepali context, particularly concerning access to education and career opportunities in nursing (Table 3).

**Table 3.** Socio-demographic characteristics of the participants.

Participant	Education	Gender	Hospital	Position	Years of experience
1	Masters	Female	Urban; Private	Registered Nurse	7-8
2	Bachelors	Female	Urban; Private	Registered Nurse	4-5
3	Masters	Female	Urban; Private	Nurse In-Charge	8-10
4	Bachelors	Female	Urban; Private	Registered Nurse	5-6
5	Masters	Female	Urban; Private	Nurse In-Charge	12-15
6	Bachelors	Female	Urban; Private	Registered Nurse	2-3
7	Masters	Female	Urban; Private	Nurse In-Charge	10-11
8	Bachelors	Female	Urban; Private	Registered Nurse	4-5
9	Masters	Female	Urban; Private	Nurse In-Charge	15-16
10	Bachelors	Female	Urban; Private	Registered Nurse	6-7
11	Masters	Female	Urban; Private	Nurse In-Charge	9-10
12	Masters	Female	Urban; Private	Nurse In-Charge	7-8

### Main findings

**Figure 1.**
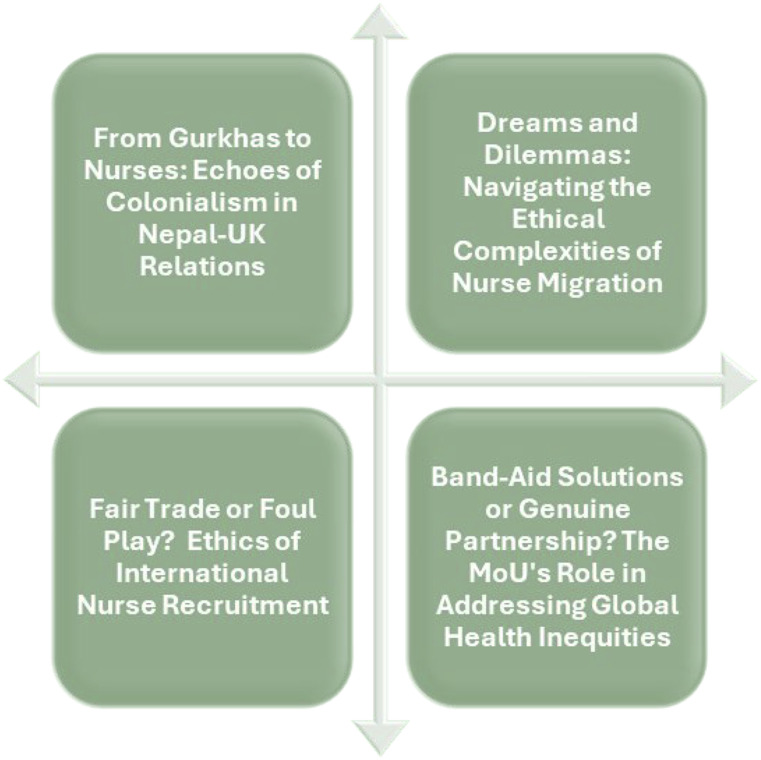
Key themes.

The results of this study generated four key themes (Figure 1) that represent nurses’ viewpoints regarding the MoU signed between the governments of Nepal and the UK.

### Theme 1: from gurkhas to nurses: echoes of colonialism in Nepal-UK relations

This theme delves into the historical and ongoing power dynamics between Nepal and the UK, highlighting how the legacy of colonialism continues to shape the perception of the nurse recruitment MoU. The nurses’ narratives reveal a sense of unease about the agreement, with concerns about potential exploitation and a lack of genuine partnership.

“It's like history repeating itself, just with different uniforms. First, it was our Gurkha soldiers fighting their wars; now, it's our nurses patching up their healthcare system. When will we stop being the resource they tap into whenever it suits them?” (P11)

This quote draws a powerful parallel between the historical recruitment of Gurkha soldiers and the current recruitment of nurses, suggesting a continuation of exploitative patterns where Nepal's human resources are utilized to serve the UK's needs.

“This MoU makes me feel like we're still under their thumb. They dictate the terms we follow. Where's the equality in that? It's time for Nepal to stand up and demand a truly fair and just partnership. Also, did the UK government forget that the Nepalese nurses had been migrating in numbers even before this agreement?” (P1)

This quote highlights the perceived power imbalance in the MoU, with nurses feeling that Nepal is not treated equally. It also points to the agency of Nepali nurses, who have been migrating independently, suggesting that the MoU may not be driven solely by their needs.

“The language of 'partnership' and 'mutual benefit' rings hollow when the power dynamics are so skewed. We're not negotiating as equals; we're being offered a deal we can't refuse […].” (P5)

This quote further emphasizes the lack of genuine partnership in the MoU, with nurses feeling that the agreement is more about the UK's needs than a mutually beneficial arrangement.

“A substantial number of resources are invested in preparing qualified nurses, from family contributions to their education at universities and the training provided by mentors at hospitals. Nurses are not developed overnight. The Nepalese government should inform its citizens about the benefits of this agreement for the country. Will the UK be investing in our hospitals or educational institutions? If not, this is not just a brain drain, but also a drain on Nepal's resources as we train nurses, equip them with skills, and then send them off to the UK.” (P7)

This quote raises concerns about the potential for “brain drain” and the loss of valuable resources invested in training Nepali nurses. It also questions the reciprocity of the agreement and whether the UK will invest in Nepal's healthcare system to mitigate the impact of nurse migration.

### Theme 2: dreams and dilemmas: navigating the ethical complexities of nurse migration

This theme explores the internal conflict experienced by Nepali nurses as they weigh their personal aspirations for professional growth and better opportunities against their ethical obligations to their communities and Nepal’s healthcare system.

“It's a constant battle between my head and my heart. My head tells me to go, to seize this chance for a better life. But my heart aches at the thought of leaving […].” (P2)

This quote poignantly captures the emotional turmoil nurses face as they grapple with the decision to migrate. The allure of a better future abroad clashes with the deep-rooted ties to their homeland and the communities they serve.

“I feel like I'm caught between two worlds. On the one hand, I'm excited about the opportunities the UK offers - better pay, more respect, a chance to grow. I am also happy that our experiences and qualifications have been recognized. But on the other hand, I'm troubled by the knowledge that my skills are desperately needed here in Nepal.” (P12)

This quote highlights the duality of the nurses’ experiences, acknowledging the potential benefits of migration while also recognizing the potential negative impact on Nepal’s healthcare system.

“I believe in the power of knowledge and experience. If I go to the UK, I can learn a lot and bring those skills back to Nepal. But will I come back? It's a gamble that I feel I have to take. There needs to be a good incentive to come back. Either Nepal will have to step up, or the UK government should mandate nurses participating in this scheme to return after gaining a few years of experience. Unless these two things happen, there is a slim chance that nurses will return.” (P7)

This quote underscores the nurses' commitment to their profession and their desire to contribute to Nepal's healthcare system, even as they consider migrating for personal and professional growth. It also raises important questions about the sustainability of the MoU and the need for policies that encourage return migration.

### Theme 3: fair trade or foul play? Ethics of international nurse recruitment

This theme delves into the heart of the ethical debate surrounding the MoU, questioning whether it represents a fair exchange or a form of exploitation disguised as a mutually beneficial agreement. The nurses’ perspectives reveal a mix of hope, skepticism, and pragmatism as they weigh the potential benefits and drawbacks of this international recruitment initiative.

“This agreement is a step in the right direction. For too long, Nepali nurses have been undervalued and underpaid, both at home and abroad. Most Nepali nurses go overseas as students and study more, even if they have the required qualifications. Having the chance to go directly as an employee with support from the UK government is a great step. I feel that this deal finally recognizes our experience and qualifications as valuable, and that's a powerful message.” (P3)

This quote reflects a sense of optimism and empowerment, with the nurse acknowledging the potential for the MoU to address the historical undervaluation of Nepali nurses and provide them with better opportunities.

“The real question is whether this MoU will lead to a more just and equitable global healthcare system. Will it empower nurses in both Nepal and the UK, or will it simply perpetuate the existing power imbalances? That's the ethical test it needs to pass.” (P12)

This quote emphasizes the ethical responsibilities of the MoU, urging it to promote a more just and equitable global healthcare system instead of merely catering to the interests of the UK.

“I wonder who benefits most from this arrangement. Is it the nurses, the government, or the hospitals that are looking to fill their staffing gaps? I am perplexed that the Nepalese government agreed to sign this deal. Is the UK investing in our hospitals, or is it giving us aid for this? This all seems odd to me, considering we already have a nurse exodus. We need to ensure that this isn't just another form of exploitation dressed up in the language of ‘fair trade’.” (P5)

This quote expresses skepticism about the MoU’s true intentions. It questions the distribution of benefits and raises concerns about potential exploitation and the lack of investment in Nepal’s healthcare system.

“I believe this deal sends a positive message to other developing countries and also puts Nepal in the spotlight. Our health workforce is capable of working in a first-world country. However, will this act as a strong incentive for future nursing students to pursue nursing because they can go to the UK? It's a tough call.” (P4)

This quote acknowledges the MoU’s potential positive impact on Nepal’s reputation and its potential to inspire future nurses. However, it also raises concerns about the agreement’s potential to exacerbate brain drain and create a cycle of dependency.

### Theme 4: band-aid solutions or genuine partnership? The MoU’s role in addressing global health inequities

This theme broadens the analysis beyond the immediate impact on Nepali nurses to consider the MoU’s implications for global health equity. The nurses’ perspectives reveal a nuanced understanding of the potential for both positive and negative consequences, highlighting the need for genuine partnership and sustainable solutions.

“I see this MoU as a potential catalyst for change. If it leads to increased investment in Nepal's healthcare system and improved working conditions for nurses here, then it could be a win-win for everyone.” (P8)

This quote reflects a sense of cautious optimism, suggesting that the MoU could be a positive force if it leads to genuine investment in Nepal's healthcare system and addresses the underlying reasons for nurse migration.

“This MoU feels like a band-aid solution. It might temporarily alleviate the UK's staffing crisis, but it does nothing to address the underlying reasons why so many Nepali nurses feel compelled to leave in the first place.” (P10)

This quote highlights the concern that the MoU may be a short-term fix for the UK’s healthcare workforce challenges, failing to address the root causes of nurse migration from Nepal.

“The MoU's success depends on how it's implemented. If it's just about filling vacancies in the UK, then it's a missed opportunity. But if it leads to genuine collaboration and investment in Nepal's healthcare system, then it could be a game-changer.” (P6)

This quote emphasizes the importance of genuine partnership and collaboration for the MoU to be successful, highlighting the need for investment in Nepal’s healthcare system and a focus on mutual benefit.

“The MoU is diverting attention from the real issues on both sides. Nepal is offering assistance despite its own declining health infrastructure and workforce. Meanwhile, the UK seems more focused on seeking help from Nepal, a country that WHO has designated as ‘red-listed’, instead of concentrating on strengthening its domestic workforce. There seems to be a lack of accountability on both sides, and this is the crux of the issue.” (P9)

This quote raises critical questions about the accountability of both the UK and Nepal in addressing the root causes of healthcare workforce challenges and the ethical implications of recruiting from a country facing its own shortages.

## Discussion

The Nepal-UK nurse recruitment MoU evokes a complex interplay of historical echoes, power imbalances, and ethical considerations. It stirs concerns about the potential perpetuation of neocolonial exploitation, particularly in the realm of healthcare ethics.^36–38^ Though positioned as a solution to workforce shortages in the UK and the upliftment of Nepali nurses, the agreement risks amplifying global inequalities by attracting skilled healthcare workers from Nepal, a country grappling with its healthcare challenges.^39^ This dynamic evokes a sense of historical injustice, reminiscent of the Gurkha soldiers who served the British Empire but were later denied settlement rights in the UK.^40^ While the MoU claims to prioritize ethical recruitment, the lived experiences of Nepali nurses highlight the potential for exploitation and a lack of transparency in the process. The power imbalance between the UK and Nepal raises concerns about whether Nepali nurses are fully informed and empowered to make decisions about migration under this agreement. Prior research on Nepali nurses in the UK has emphasized the ethical concerns regarding the exploitation of migrant nurses. Nepali migrant nurses, having high qualifications and extensive experience in critical care, management, and education, found themselves working in a residential care facility, a role that migrant nurses often refer to as “British Bottom Care (BBC).”^41^ Moreover, the potential “brain drain” from Nepal, a “red-listed” country by the WHO,^16^ is facing critical health workforce challenges.^42^ The exodus of skilled nurses further strains Nepal’s healthcare system, leaving behind those most needing care.^2^ While some nurses view the MoU as a step towards recognizing their value and offering them better opportunities, the overarching concern remains: is this truly a fair exchange, or is Nepal once again bearing the brunt of a system prioritizing the needs of wealthier nations?

The narratives of Nepali nurses reveal a poignant internal conflict between their aspirations and their sense of ethical responsibility towards their communities and the national healthcare system. This internal struggle is further compounded by the stark realities of Nepal’s healthcare landscape, where limited opportunities, low wages, workplace violence, and a lack of recognition often push nurses towards seeking greener pastures abroad.^2,43^ While the MoU offers a seemingly attractive pathway to fulfill personal dreams and ambitions, it also triggers a moral obligation towards those left behind. From an ethical perspective, the question of whether nurses should prioritize collective well-being over individual aspirations is a complex one. While it is undeniable that the migration of skilled healthcare professionals can harm source countries,^44^ it is equally important to recognize the individual’s right to pursue a better life and professional fulfillment.^19^ The ethical dilemma lies in finding a balance between these competing interests. As our study reveals, Nepali nurses are acutely aware of the potential impact of their departure on Nepal’s healthcare system. However, they also express a strong desire to gain experience and knowledge abroad, hoping to eventually return to contribute to their home country. This highlights the potential for “brain gain,” where migration can lead to acquiring new skills and knowledge to benefit the source country in the long run.^45^ While the MoU facilitates nurse migration, it is crucial to recognize that it does not directly address the systemic issues within Nepal’s healthcare system that contribute to the push factors for migration. It is, therefore, imperative to distinguish between foreign recruitment of nurses and the free choice of nurses to migrate.

While upholding the autonomy of Nepali nurses to pursue opportunities abroad, the potential for discriminatory or coercive hiring practices rooted in systemic inequalities and the exploitation of nurses from developing countries necessitates careful consideration.^46–48^ The absence of enforceable ethical standards and the stark economic disparities between source and destination countries further exacerbate this issue.^38,49,50^ Although the Nepal-UK MoU aims to address these challenges by establishing a framework for ethical recruitment, prohibiting migration agent fees, and safeguarding the rights of Nepali healthcare professionals,^26^ its effectiveness hinges on robust implementation and enforcement. The historical context of discriminatory practices against foreign nurses, particularly those from non-European and non-White countries, underscores the need for vigilance in upholding ethical standards.^46–48^ This includes addressing systemic biases in language tests, devaluing migrant nurses’ experiences, qualifications, and regulatory barriers that create structural racism and hinder career progression for migrant nurses.^47,48^

Furthermore, the aggressive recruitment of nurses from LMICs such as Nepal, often driven by cost considerations rather than genuine partnership or a sustainable domestic workforce, raises ethical concerns about the depletion of healthcare resources in source countries and the perpetuation of global health inequalities.^6,11,19,51^ The incongruity between the rhetoric of global solidarity and the reality of self-interested actions further complicates the ethical landscape.^52^ While espousing ideals of a borderless world and global citizenship, high-income countries often prioritize national interests when faced with domestic challenges, revealing a deeply ingrained nationalist and arguably neocolonial mindset plaguing global health equity.^53^ The MoU’s commitment to collaborate on increasing healthcare capacity in Nepal offers a glimmer of hope, but its success in mitigating these complex ethical challenges and fostering a truly equitable global health workforce remains to be seen.

### Strengths, limitations, and future research

This study makes several key contributions to the research field. First, it provides a unique and timely analysis of the ethical implications of the Nepal-UK nurse recruitment MoU, a policy initiative with potentially far-reaching consequences for both countries. By centering the voices of Nepali nurses, the study offers a nuanced understanding of the complex interplay between individual aspirations, collective responsibilities, and global health equity considerations. Second, the study challenges simplistic narratives surrounding international nurse recruitment, highlighting the need for a more critical and ethically grounded approach that moves beyond the rhetoric of “fair trade” and acknowledges the historical power imbalances and potential for exploitation. Third, the findings expose the ethical dilemmas nurses face as they navigate the decision to migrate, underscoring the need for policies that support both individual aspirations and encourage investment in source countries’ healthcare systems. Finally, the study contributes to the growing body of literature on neocolonialism in healthcare, demonstrating how historical power dynamics continue to shape contemporary relationships between high-income and low- and middle-income countries.

While offering valuable insights into the ethical complexities surrounding the Nepal-UK nurse recruitment agreement, this study acknowledges certain limitations. The sample size of 12 nurses, while providing rich qualitative data, may not fully capture the diversity of perspectives within the broader Nepali nursing community. Additionally, the focus on nurses in urban settings and all female participants limits the generalizability of the findings to other regional and rural areas of Nepal. Further research is warranted to explore the views of nurses from different geographical and socio-economic backgrounds and those with varying levels of professional experience and career aspirations. Future studies could also investigate the perspectives of Nepali nurses who have migrated to the UK under this agreement, providing a longitudinal analysis of their experiences and the MoU’s impact on their lives and careers. Moreover, a comparative study exploring nurses’ perspectives from other source countries involved in similar bilateral agreements could offer valuable insights into the global dynamics of nurse migration and ethical recruitment practices.

## Recommendations and conclusion

The study’s findings recommend that policymakers in Nepal and the UK should engage in transparent and equitable dialogue with Nepali nurses. This approach aims to address their concerns and ensure that their voices are heard in both the implementation and ongoing evaluation of the MoU. Moreover, it is imperative to invest in strengthening Nepal’s healthcare system, improving working conditions for nurses, and creating opportunities for professional development to mitigate the push factors driving migration. The UK, on its part, should focus on addressing its internal healthcare workforce challenges and reducing its reliance on international recruitment.

This qualitative study has illuminated the multifaceted ethical landscape surrounding the Nepal-UK nurse recruitment MoU as perceived by Nepali nurses. Their voices reveal a complex interplay of aspirations and anxieties, highlighting the tensions between individual opportunities and collective well-being, historical power imbalances, and the potential for neocolonial exploitation. This study serves as a call for a more ethical and sustainable approach to global nurse migration. By prioritizing fairness, transparency, and mutual benefit, we can create a system that empowers nurses, strengthens healthcare systems, and upholds the principles of global health equity.
